# Development and Physicochemical Stability of an Oral GS‐441524 Suspension for Weight‐Adjusted Treatment of Feline Infectious Peritonitis

**DOI:** 10.1002/vms3.71073

**Published:** 2026-06-30

**Authors:** Cédric Wernli, Ursula Streit

**Affiliations:** ^1^ Private University in the Principality of Liechtenstein (UFL) Triesen Liechtenstein; ^2^ Lehenmatt‐Apotheke AG Basel Switzerland; ^3^ Dr. E. Graeub AG Bern Switzerland

**Keywords:** cats, feline infectious peritonitis, GS‐441524, oral suspension, pysicochemical stability, veterinary compounding

## Abstract

**Background:**

GS‐441524 is a key antiviral agent for the treatment of feline infectious peritonitis (FIP), yet no authorized veterinary medicinal product is currently available.

**Objectives:**

To provide a quality‐assured alternative to unregulated products, we developed a 50 mg/mL oral suspension and evaluated its physicochemical stability.

**Methods:**

Three independent batches were prepared using Syrspend SF pH 4.0 and a veterinary flavouring agent. Stability was assessed at 5°C, 25°C/60% RH and 40°C/75% RH (transport simulation) over 12 weeks. In‐use stability was evaluated for 28 days following repeated withdrawals. Stability was defined as maintenance of drug concentration within 90%–110% of initial values (45–55 mg/mL), pH 3.5–4.5 and relative density 1.02–1.04 g/mL.

**Results:**

All batches remained within predefined acceptance limits under all conditions. The formulation, therefore enables accurate oral dosing of GS‐441524 for weight‐adjusted therapy and provides a regulated alternative to unlicensed products.

**Conclusions:**

These findings support the suitability of the formulation as an extemporaneously compounded preparation under veterinary supervision. Pharmacokinetic and clinical performance were not assessed and should be addressed in future studies to further substantiate therapeutic use.

## Graphical abstract

A 50 mg/mL oral GS‑441524 suspension for feline infectious peritonitis (FIP) was developed and shown to remain physico‐chemically stable for at least 12 weeks under tested storage conditions and 28 days in‑use, supporting accurate weight‑adjusted dosing as an extemporaneously compounded formulation under veterinary supervision.

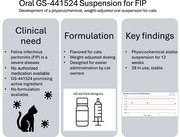

## 1 Introduction

Feline coronavirus (FCoV) is an RNA virus with a high prevalence in cat populations. The prevalence of FCoV was tested in several countries using faecal samples or rectal swabs, whereas the RNA of the virus could be detected in 31.8%–100% (Klein‐Richers et al. 2020). However, specific global prevalence data are rare (Mir et al. 2025). Living conditions such as sharing litter boxes and crowded living, shared feeding boxes are discussed as predisposing factors, but the studies to confirm those factors are limited (Klein‐Richers et al. 2020; Thayer et al. 2022). In one study, the prevalence of FCoV in 37 German catteries with 179 cats was investigated, where none of the catteries were free of FCoV (Thayer et al. 2022). Serological data suggest that 25% of domestic (nonpurebred) cats in pet households and 75%–100% of purebred cats in catteries have antibodies against FCoV (Rohrbach et al. 2001). A small proportion of cats infected with FCoV becomes severely ill with this infection after mutation of the low‐virulence FCoV in the carrier animal itself combined with host‐depended factors such as immune response and genetic susceptibility and develops feline infectious peritonitis (FIP). In one study including 73 multi‐ and single‐cat households, 22 of 282 seropositive kittens (7.8%) subsequently developed FIP. At this time, no treatment was available and the mortality rate for cats was greater than 95% (Rohrbach et al. 2001). The mechanisms underlying viral mutation and the factors determining why some cats develop FIP while others remain asymptomatic are not yet fully understood (Mir et al. 2025). Familial patterns, breed predispositions and associations with reduced heterozygosity indicate a heritable component for developing FIP. Inbreeding and the use of breeding animals linked to FIP cases are discouraged. Environmental factors, including stress and multi‐cat housing, further increase risk by promoting FCoV transmission, particularly in settings with high population density, frequent mixing of cats, chronic shedders or inadequate sanitation (Kennedy 2020). In one study, they concluded that affected cats were more likely to be young, purebred and intact males, whereas spayed females and survivors were less frequent than in controls (Rohrbach et al. 2001).

FIP is one of the most important infectious diseases in cats, and virtually every practicing small animal veterinarian will see cases. It is estimated that around 0.3%–1.4% of deaths in veterinary institutions are due to infection with FCoV and the resulting disease FIP (Thayer et al. 2022). These numbers are an estimation based on investigation of 651 cats at first diagnosis of FIP and 89 control cats that died or were euthanized during a 10‐year period in a veterinary medical teaching hospital in North America (Rohrbach et al. 2001).

Diseases such as Ebola and Middle East Respiratory Syndrome have led to intensive research into inhibiting the replication of RNA viruses. The prodrug GS‐5734 (remdesivir, Gilead Sciences) prevented Ebola infections in rhesus monkeys, and the molecule also showed success in coronavirus infections in tissue and mouse experiments. These findings led to intensified research into this molecule and its active metabolite GS‐441524 for the treatment of FIP in cats. It was shown that the molecule GS‐441524, with its molecular weight of < 900 Da and size of less than 1 nm, can penetrate the cell and directly interfere with the cellular processes of viral replication as a nucleoside analogue. In an initial study involving 31 cats with FIP, parenteral treatment with GS‐441524 demonstrated promising efficacy and an acceptable safety profile treatment for FIP (Pedersen et al. 2019). In a study involving 307 cats suffering from FIP that were treated parenterally and orally with remdesivir and GS‐441524 from a legal source with a known concentration, 84.4% of the cats survived the disease (Taylor et al. 2023). The recommended duration of therapy with the oral formulation of GS‐441524 in the literature was 84 days. A prospective randomized controlled study investigated whether shortening the duration of therapy to 42 days would be sufficient to effectively treat the infection. Forty cats with FIP were included in the study, all of which received 15 mg/kg GS‐441524 orally every 24 h for 42 or 84 days. The observation period was 168 days after the start of therapy. Thirty‐eight cats recovered from the infection (19 cats with 42 days and 19 cats with 84 days of therapy). At the end of the observation period, all 38 cats were in full remission. Based on these data, a treatment duration of 42 days (6 weeks) was suggested, as this is associated with lower treatment costs, a reduced risk of side effects and a lower potential risk of resistance development; however, the study enrolled only 40 cats and had a maximum follow‐up of 168 days, and further studies with larger populations and longer follow‐up are needed to confirm these findings and validate shortened treatment regimens (Zuzzi‐Krebitz et al. 2024).

The lack of approval and availability of the active ingredient GS‐441524 led to owners of infected animals that were not allowed to participate in studies obtaining the drug on the black market. Support groups were formed on social media (with names such as ‘FIP Warriors’). However, the questionable origin of these illegal drugs also carries considerable risks. In a study on the quality of preparations available on the black market, 87 parenteral injection formulations were examined, 95% of which contained a higher concentration of GS‐441524 (on average 39% higher) than declared. The average pH value measured (pH 1.30) was also lower than the physiologically recommended pH value for parenteral preparations for subcutaneous injection. Of the 40 oral formulations (tablets and capsules) examined, 43% (on average 75% higher) showed a higher concentration of GS‐441524, while 58% of the preparations examined contained less (on average 39% less) active ingredient than declared (Kent et al. 2024). No medicinal product has been available in Switzerland to date. Only in the context of clinical studies at the University Hospitals of the Vetsuisse Faculties in Zurich and Bern were the 50 mg GS‐441524 tablets manufactured by BOVA Specials, London, United Kingdom, available. These tablets are not approved, but are legally manufactured and used for study purposes only.

In Switzerland, extemporaneous compounding is permitted when no authorized medicinal product exists for a serious condition and when quality‐assured preparation is required. The present work addresses this gap by developing a stable oral GS‐441524 suspension suitable for veterinary prescription and supervised outpatient use. The aim of this study was to formulate a liquid oral preparation that enables accurate weight‐adjusted dosing according to established regimens (e.g., 15 mg/kg once daily) and can be administered easily by pet owners under veterinary guidance.

## 2 Materials and Methods

### 2.1 Materials

The active ingredient GS‐441524 was obtained from Senova Technology Co. Ltd., Nanshan District of Shenzhen, China, and its quality was confirmed by additional analysis from an independent European GMP‐certified laboratory, whereas the identity, residues and impurities were confirmed as stated on the certificate of analysis from the manufacturer. Due to its poor water solubility of 0.105 mg/mL (Wei et al. 2021) and failed attempts to use beta‐cyclodextrin as a solubilizer, it was decided to produce a suspension for oral administration. Syrspend SF pH 4.0 from Fagron GmbH, Glinde, Germany, was chosen as the basis for the suspension, which, according to the company's specifications, contains purified water, modified corn starch, sodium citrate, citric acid, sucralose, sodium benzoate, malic acid and simethicone. In addition, a liquid flavouring agent from FlavorRx, Columbia, Maryland, USA, with the flavour ‘Chicken Pot Pie’ was used for flavouring. This flavouring agent was developed especially for animals, has been approved by the FDA, and is considered safe. The suspension was filled into 30 mL white vials and sealed with a tamper‐evident adapter for dosing syringes. The dispensing containers, including closures, were ordered from E. Anwander & Cie. AG, Oberwil, Switzerland.

### 2.2 Preparation of the Suspension

The concentration of the suspension and the dosage were adjusted to the expected therapy. Due to a lack of data on the stability and shelf life of GS‐441524 in the suspension base with the flavouring agent, a stability study was conducted. No Data were collected and found concerning the chemical compatibility of GS‐441524 and the individual components of Syrspend SF pH 4.0 beyond the physicochemical stability data presented in this study.

The three independent batches of 50 mg/mL GS‐441524 used for the stability study were prepared as follows:

Place approximately 80% Syrspend SF pH 4.0 in a beaker, add GS‐441524 and FlavorRx ‘Chicken Pot Pie’ at 3%, homogenize with a magnetic stirrer at 600 rpm and add the remainder of Syrspend SF pH 4.0. Then homogenize the suspension again on the magnetic stirrer and transfer to a dispensing vessel. The suspension was filled into white glass bottles and labelled, ensuring protection from light. During the study, the bottles were stored in light‐proof, climate‐controlled cabinets. Formal photostability testing in accordance with ICH Guidelines was not performed, which represents a limitation of this study.

### 2.3 Stability Study Design

Stability of unopened bottles for storage in calibrated heat or refrigerated cabinets.

The concentration of the active ingredient, pH value and density of the GS‐441524 suspension were measured in all three batches as follows:

Measurement points: T0 (1 day after manufacturing the suspension), Weeks 1, 5 and 12 at 5°C (±3°C) and 25°C, 60% relative humidity (RH), as well as additionally on Days T0, 3 and 10 at 40°C, 75% RH (stability for sample transport). This temperature was intended to simulate transport stress and should not serve as a formal accelerated stability study as described in ICH guidelines.

### 2.4 Stability of Opened Bottles for Storage During Use by the Animal Owner

The concentration of the active ingredient, pH value and density of the GS‐441524 suspension were measured in all three batches, which had already been stored closed for 5 weeks, after repeated opening and removal of at least 0.1 mL over the period, as follows:

Measurement points: 14 days open (with 13 withdrawals), 28 days (with 25 withdrawals) at 5°C (± 3°C) and 25°C, 60% RH.

Stability was defined as maintenance of GS‐441524 concentration within 90%–110% of the initial value (45–55 mg/mL), with pH remaining between 3.5 and 4.5 and relative density between 1.02 and 1.04.

Sample preparation was performed based on the publication by Geiger et al. (2018) for determining the stability of sildenafil citrate in Syrspend.

### 2.5 Analytical Methods

Standard for determining the calibration curve.

Place 10 mg GS‐441524 in a 20 mL volumetric flask, add 2 mL dimethyl sulfoxide (DMSO) and dissolve. Make up the solution to 20 mL with acetonitrile (ACN). Add 1 mL of this solution to a 20 mL measuring flask and make up to 20 mL with 20 mM ammonium acetate buffer, pH 4.5 (25 µg/mL GS‐441524). A multipoint calibration was performed to calculate the concentrations.

The standard (25 µg/mL GS‐441524) was prepared and measured with each measurement run. The standard was injected as both the first and last sample in each run. These measurements were used to assess the stability of the method. The concentrations were calculated using linear regression to determine the linearity of the method.

### 2.6 Extraction of Samples From the Suspension

Place 1.03 g of oral suspension with GS‐441524 at 50 mg/mL in a 10 mL volumetric flask, add DMSO and dissolve completely. Transfer 1 mL of this solution to a 10 mL volumetric flask and make up to 10 mL with ACN. Transfer 1 mL of this solution to a 20 mL volumetric flask and make up to 20 mL with 20 mM ammonium acetate buffer, pH 4.5 (with a density of 1.03 g/mL, a concentration of 25 µg/mL GS‐441524 is obtained).

The samples were measured based on the publication by Kimble et al. (2023) on the determination of GS‐441524 in feline plasma using HPLC.

Concentrations were measured using reversed‐phase HPLC with DAD detection. The measurement was performed using an Agilent HPLC, Model 1260, with the following parameters:

Column: Zorbax SB C18, 5 µm, 150 mm × 4.6 mm, column temperature: 35°C, sampler temperature: 20°C, injection volume: 10 µL, detection using DAD at 241 nm.

Mobile phase: 20 mM ammonium acetate buffer (adjusted to pH 4.5 with acetic acid) for Mobile Phase A with 5% ACN and for Mobile Phase B with 70% ACN. Gradient 0–1 min (0%–5% B), 1–2 min (5% B), 2–5 min (5%–50% B), 5–10 min (50%–90% B), 10–11 min (90% B) and 11–12 min (90%–0%) at a flow rate of 1.2 mL/min and a total run time of 15 min.

The pH value was measured using a calibrated Seven Easy pH meter from Mettler Toledo (measuring method: potentiometry), and the density was measured using a calibrated Easy D40 densitometer from Mettler Toledo (measuring method: oscillating U‐tube method).

For each batch, measurements were performed in duplicate. Mean values and standard deviations (SDs) were calculated. Linear regression analysis was used to assess potential trends in concentration over time.

Formal analytical method validation, including determination of recovery, precision and accuracy in the suspension matrix, was not conducted as part of this study. Results should be interpreted in the context of this limitation.

Potential adsorption of GS‐441524 to the inner glass surface of the dispensing vial was not assessed and cannot be excluded.

It is acknowledged that formal preservative efficacy testing of sodium benzoate, as required by pharmacopoeial standards, was not conducted as part of this study.

### 2.7 Use of Artificial Intelligence Tools

Microsoft Copilot and DeepL were used solely to assist with language refinement during manuscript preparation. No artificial intelligence tool was used to generate, analyse or interpret data, nor to create scientific content. All study design, methods, results and conclusions were developed and verified entirely by the authors.

## 3 Results

The method was linear from 1 to 50 µg/mL with an *R* ^2^ of 0.9995. The chromatograms showed no additional peaks throughout the entire measurement period.

Table 1 shows the mean values of the measurements of the three batches of pH, density and concentration of GS‐441524 in the suspension, which were stored at different temperatures. Table 2 shows the mean values obtained during the 28 day in‐use stability study.

**TABLE 1:** Mean values of active ingredient concentration, pH value and density from three batches of the GS‐441524 suspension in closed containers under different temperature conditions for stability testing.

	pH value	SD	Relative density	SD	Concentration GS‐441524 (mg/mL)	SD
	Mean		Mean		Mean	
Time point, *T* = 0	3.91	0.02	1.0335	0.001	50.82	0.35
Temperature						
5°C (±3°C)	pH value		Relative density		Concentration GS‐441524 (mg/mL)	
Time point	Mean	SD	Mean	SD	Mean	SD
Weeks						
1	4.03	0.01	1.031	0.001	51.04	0.47
5	4.01	0.00	1.033	0.000	50.92	0.77
12	3.99	0.01	1.033	0.000	50.69	0.53
Temperature						
25°C/60% RH	pH value		Relative density		Concentration GS‐441524 (mg/mL)	
Time point	Mean	SD	Mean	SD	Mean	SD
Weeks						
1	4.01	0.00	1.032	0.001	51.22	0.08
5	4.01	0.01	1.033	0.002	51.33	0.90
12	4.00	0.02	1.034	0.001	50.52	1.34
Temperature						
40°C/75% RH	pH value		Relative density		Concentration GS‐441524 (mg/mL)	
Time point	Mean	SD	Mean	SD	Mean	SD
Days						
3	3.92	0.01	1.033	0.002	50.75	0.65
10	4.04	0.01	1.033	0.001	51.37	0.43

**TABLE 2:** Mean values for active ingredient concentration, pH value and density from three batches of the GS‐441524 suspension after multiple sampling of at least 0.1 mL for in‐use stability testing over 28 days.

Temperature	In‐use stability					
5°C (±3°C)	pH value		Relative density		Concentration GS‐441524 (mg/mL)	
Time point (days after opening)	Mean	SD	Mean	SD	Mean	SD
0	4.01	0.00	1.033	0.000	50.92	0.77
14	4.02	0.01	1.033	0.000	51.07	0.39
28	4.00	0.01	1.032	0.001	51.29	0.57
						

Temperature	In‐use stability					
25°C/60% RH	pH value		Relative density		Concentration GS‐441524 (mg/mL)	
Time point (days after opening)	Mean	SD	Mean	SD	Mean	SD
0	4.01	0.01	1.033	0.002	51.33	0.90
14	4.00	0.00	1.034	0.000	51.93	0.25
28	4.00	0.01	1.032	0.002	52.17	1.13

**FIGURE 1:**
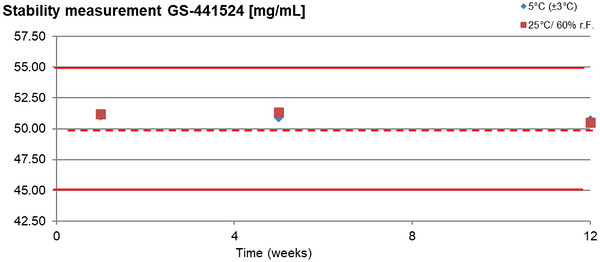
Graphical representation of the mean values across three batches of concentration measurements of the GS‐441524 suspension at 5°C and 25°C/60% RH.

**FIGURE 2:**
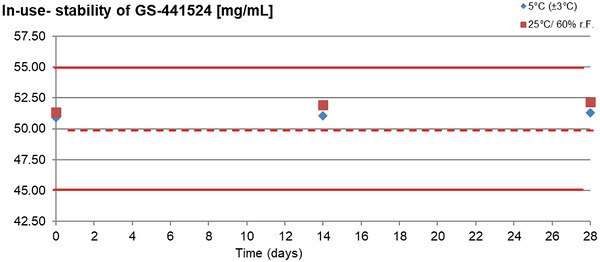
Graphical representation of the mean values of the concentration measurements from three batches of the GS‐441524 suspension at 5°C and 25°C/60% RH over 28 days. Day 0 is the first measuring point after opening.

Figure 1 shows the mean values obtained for the two temperatures of 5°C and 25°C/60% RH, while Figure 2 shows those of the in‐use stability measurement.

**FIGURE 3:**
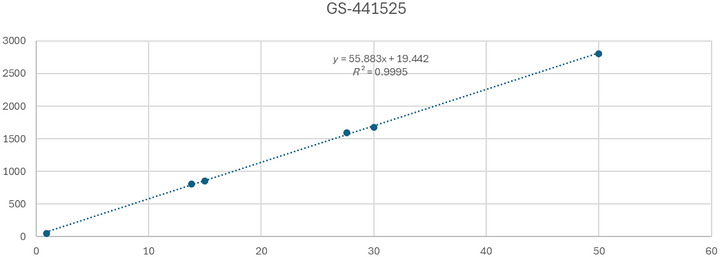
Linearity of the method for the GS‐441524's measurement range of 1–50 µg/mL.

Figure 3 shows the linearity of the method for the GS‐441524's measurement range of 1–50 µg/mL.

**FIGURE 4:**
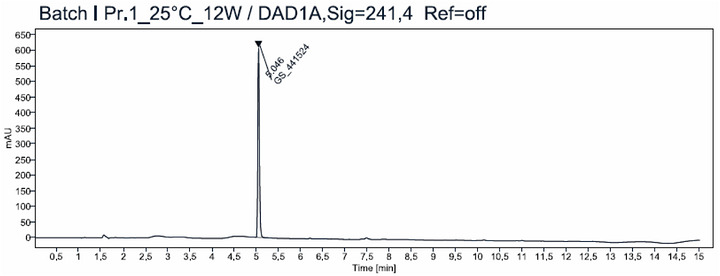
Chromatogram of Batch 1 after 12 weeks of storage at 25°C.

Figure 4 shows the chromatogram of Batch 1 after 12 weeks of storage at 25°C.

## 4 Discussion

The present results show a stable liquid formulation of a GS‐441524 suspension in a sealed container at room temperature, limited for a time period over 12 weeks. Further stability of the suspension needs to be assessed. The concentration of all three batches shows no significant variation over the observed period of 12 weeks. All values are within the defined acceptance values. Even repeated removal of the suspension over several days has no influence on the stability of the concentration over the observation period of 28 days. This period of time can be considered sufficient for the required duration of therapy and suspension content in the bottle. The measured values at 40°C/75% RH over 10 days show no significant change in the amount of GS‐441524 over this time period. Since shipping only takes place within Switzerland, the observation period was considered sufficient; however, fluctuations of temperature, which may occur were not assessed in this study and those data cannot replace a formal transport stability study.

The 50 mg/mL concentration allows practical, weight‑adjusted dosing consistent with published therapeutic regimens (e.g., 15 mg/kg once daily for 42–84 days), resulting in typical administration volumes of 0.8–1.5 mL for most cats. Stable concentration over 12 weeks may help to reproducible exposure and may help to reduce the risk of under‑ or overdosing, which is particularly relevant given the variability reported in unregulated GS‑441524 products.

The measurements at 5°C and at 25°C/60% RH show comparable values, indicating that the suspension can be stored at room temperature and no instability is to be expected. Both the pH value and the relative density remain stable over the observed period.

Due to a lack of published data on the formulation and stability of the active ingredient GS‐441524, no comparative values could be found, which is why the chosen formulation is based on empirical values from other suspensions using Syrspend SF pH 4.0.

The available data on the formulation refer solely to stability in the selected medium. Pharmacokinetic data were not collected and would have to be collected if approval of this preparation were sought. Likewise, no clinical studies were conducted on cats. While regulatory frameworks differ internationally, extemporaneous compounding remains an important mechanism to provide quality‐assured therapy when no authorized product exists. The present formulation offers a regulated alternative that aligns with veterinary compounding standards, without implying equivalence to an authorized medicinal product.

### 4.1 Limitations

The following limitations of the present study are acknowledged:

### 4.2 Microbiological Stability

Microbiological stability has not been determined. The suspension base Syrspend pH 4.0 contains sodium benzoate as a preservative. Kegele et al. published studies in which antimicrobial efficacy testing (AET) was conducted in accordance with US Pharmacopeia guidelines. They were able to demonstrate for the tested microorganisms (*Candida albicans*, *Aspergillus brasiliensis*, *Escherichia coli*, *Pseudomonas aeruginosa* and *Staphylococcus aureus*) that the microbial counts remained below the limit throughout the 14‐ and 28‐day observation periods (Kegele et al. 2025a, 2025b, 2025c, 2025d, 2025e). These data support the stability of the suspension base over the observed time period; however, microbial stability must be tested with the active ingredient GS‐441524 to verify this aspect.

### 4.3 Physical Stability

No physical stability measurements were performed, including measurements of sedimentation rate, redispersibility or viscosity. These data are important for assessing the uniformity of dosage and should be included in future studies. Visser et al. (2018) investigated the rheological and sedimentation properties of Syrspend(R) SF pH 4.0 with paracetamol and were able to demonstrate that a stable suspension is obtained, with Syrspend(R) SF pH 4.0 exhibiting higher viscosity than the compared suspension, which is preferable in terms of sedimentation. This study is based on paracetamol and not on the active ingredient GS‐441524 under investigation; however, it demonstrates the fundamental properties of the base formulation used.

### 4.4 Adsorption

The potential for GS‐441524 to be absorbed through the glass lining of the vessel has not been investigated and cannot be ruled out. Given the stable concentrations observed over the measured period, we consider this risk to be low; however, this would need to be determined in further studies.

### 4.5 Photostability

No study has been conducted to determine the photostability of the active ingredient GS‐441524. The storage container is white and has a label affixed to it, which minimizes exposure to light. Nevertheless, this characterization has not been performed and would need to be conducted in further studies.

### 4.6 Analytical Method Validation

The HPLC measurement method has not been fully validated and would need to be fully validated in further studies (including recovery, precision and accuracy). Nevertheless, the linearity and stability of the measurement results indicate that this is a reliable measurement method.

### 4.7 Shelf Life

The specific time frame for the stability study is limited to a period of 12 weeks. Further studies are necessary to assess a longer period.

### 4.8 Pharmacokinetic and Clinical Data

No pharmacokinetic studies or clinical trials have been conducted in cats. The measured physicochemical stability is not a substitute for clinical data (bioavailability, efficacy or safety), which is why these parameters would need to be determined in further studies in order to evaluate them.

## 5 Conclusions

This study demonstrates that a 50 mg/mL oral GS‐441524 suspension remains physico‐chemically stable for at least 12 weeks under the storage conditions evaluated and maintains 28‐day in‐use stability following repeated withdrawals. The formulation enables accurate and reproducible oral dosing for FIP therapy and provides a regulated alternative to unlicensed products. These findings support its use as an extemporaneously compounded preparation under veterinary supervision. With the data now available, it is possible to offer a legal, stable formulation in Switzerland for cats suffering from FIP. The formulation is easy for pet owners to administer, so that the cat can be treated in its familiar environment, following veterinary instructions, provided that its clinical condition allows this. Pharmacokinetic and clinical data were not collected as part of this study, and no clinical conclusions can be drawn from the stability data alone. Future work should include full analytical method validation, microbiological stability testing, physical stability assessment (including sedimentation, viscosity and redispersibility), photostability testing and pharmacokinetic and clinical evaluation in cats, to further support evidence‐based therapeutic use of this formulation.

## Author Contributions

**Cédric Wernli**: conceptualization, investigation, writing – original draft, methodology, validation, visualization, project administration, resources, writing – review and editing, data curation. **Ursula Streit**: investigation, writing – review and editing, visualization, formal analysis, data curation, supervision, validation, methodology, project administration, resources.

## Funding

The authors have nothing to report.

## Ethics Statement

The authors have nothing to report.

## Conflicts of Interest

Cédric Wernli is the owner of the Lehenmatt‐Apotheke AG, and Ursula Streit is employed by Dr. E. Graeub AG. These affiliations represent potential economic interests. However, they did not influence the design, conduct or reporting of this study.

## Data Availability

The data that support the findings of this study are available on request from the corresponding author. The data are not publicly available due to privacy or ethical restrictions.

## References

[vms371073-bib-0001] Geiger, C. M. , B. Sorenson , and P. Whaley . 2018. “Stability of Sildenafil Citrate Oral Suspension With Syrspend® Sf.” European Pharmaceutical Journal 65: 31–35. 10.2478/afpuc-2018-0007.

[vms371073-bib-0002] Kegele, C. S. , E. Dijkers , and H. Polonini . 2025a. “Antimicrobial Effectiveness and Physicochemical Stability of Compounded Psychiatric and Neurological Drug Suspensions in SyrSpend® SF PH4.” International Journal of Pharmaceutical Compounding 29: 319–327. 40961477

[vms371073-bib-0003] Kegele, C. S. , E. Dijkers , and H. Polonini . 2025b. “Antimicrobial Stability, pH, and Physical Appearance of Cardiovascular Drug Suspensions in SyrSpend® SF PH4: A Complementary Evaluation.” International Journal of Pharmaceutical Compounding 29: 310–318. 40961476

[vms371073-bib-0004] Kegele, C. S. , E. Dijkers , and H. Polonini . 2025c. “Microbiological and Physical Stability of Extemporaneous Oral Suspensions Compounded With SyrSpend® SF PH4.” International Journal of Pharmaceutical Compounding 29: 409–419. 41115035

[vms371073-bib-0005] Kegele, C. S. , E. Dijkers , and H. Polonini . 2025d. “Physicochemical and Microbiological Stability of Commonly Prescribed APIs in SyrSpend® SF PH4: A Comprehensive Compatibility Study.” International Journal of Pharmaceutical Compounding 29: 222–238. 40582006

[vms371073-bib-0006] Kegele, C. S. , E. Dijkers , and H. Polonini . 2025e. “SyrSpend® SF PH 4 as a Safe Vehicle for Extemporaneous Oral Suspensions: Microbiological Stability of Seven APIs.” International Journal of Pharmaceutical Compounding 29: 420–429. 41115036

[vms371073-bib-0007] Kennedy, M. A. 2020. “Feline Infectious Peritonitis: Update on Pathogenesis, Diagnostics, and Treatment.” Veterinary Clinics of North America Small Animal Practice 50: 1001–1011. 10.1016/j.cvsm.2020.05.002. 32563530

[vms371073-bib-0008] Kent, A. M. , S. Guan , N. Jacque , W. Novicoff , and S. J. M. Evans . 2024. “Unlicensed Antiviral Products Used for the At‐Home Treatment of Feline Infectious Peritonitis Contain GS‐441524 at Significantly Different Amounts Than Advertised.” Journal of the American Veterinary Medical Association 262: 489–497. 10.2460/javma.23.08.0466. 38324994 PMC11983332

[vms371073-bib-0009] Kimble, B. , S. J. Coggins , J. M. Norris , M. F. Thompson , and M. Govendir . 2023. “Quantification of GS‐441524 Concentration in Feline Plasma Using High Performance Liquid Chromatography With Fluorescence Detection.” Veterinary Quarterly 43: 1–9. 10.1080/01652176.2023.2246553. PMC10438854 37556736

[vms371073-bib-0010] Klein‐Richers, U. , K. Hartmann , R. Hofmann‐Lehmann , et al. 2020. “Prevalence of Feline Coronavirus Shedding in German Catteries and Associated Risk Factors.” Viruses 12: 1000. 10.3390/v12091000. 32911718 PMC7551668

[vms371073-bib-0011] Mir, S. , M. Peters , G. Penny , A. Agsaoa , and M. Mir . 2025. “From Challenge to Cure: A Look at Feline Infectious Peritonitis and Emerging Treatment Strategies and Breakthroughs.” Veterinary Sciences 12: 650. 10.3390/vetsci12070650. 40711310 PMC12299834

[vms371073-bib-0012] Pedersen, N. C. , M. Perron , M. Bannasch , et al. 2019. “Efficacy and Safety of the Nucleoside Analog GS‐441524 for Treatment of Cats With Naturally Occurring Feline Infectious Peritonitis.” Journal of Feline Medicine and Surgery 21: 271–281. 10.1177/1098612X19825701. 30755068 PMC6435921

[vms371073-bib-0013] Rohrbach, B. W. , A. M. Legendre , C. A. Baldwin , D. H. Lein , W. M. Reed , and R. B. Wilson . 2001. “Epidemiology of Feline Infectious Peritonitis Among Cats Examined at Veterinary Medical Teaching Hospitals.” Journal of the American Veterinary Medical Association 218: 1111–1115. 10.2460/javma.2001.218.1111. 11318361

[vms371073-bib-0014] Taylor, S. S. , S. Coggins , E. N. Barker , et al. 2023. “Retrospective Study and Outcome of 307 Cats With Feline Infectious Peritonitis Treated With Legally Sourced Veterinary Compounded Preparations of Remdesivir and GS‐441524 (2020‐2022).” Journal of Feline Medicine and Surgery 25: 1098612X231194460. 10.1177/1098612X231194460. PMC10812036 37732386

[vms371073-bib-0015] Thayer, V. , S. Gogolski , S. Felten , K. Hartmann , M. Kennedy , and G. A. Olah . 2022. “2022 AAFP/EveryCat Feline Infectious Peritonitis Diagnosis Guidelines.” Journal of Feline Medicine and Surgery 24: 905–933. 10.1177/1098612X221118761. 36002137 PMC10812230

[vms371073-bib-0016] Visser, J. C. , I. E. J. Ten Seldam , I. J. van der Linden , et al. 2018. “Comparison of Rheological and Sedimentation Behavior of Commercially Available Suspending Vehicles for Oral Pharmaceutical Preparations.” International Journal of Pharmaceutical Compounding 22: 247–251. 29878892

[vms371073-bib-0017] Wei, D. , T. Hu , Y. Zhang , et al. 2021. “Potency and Pharmacokinetics of GS‐441524 Derivatives Against SARS‐CoV‐2.” Bioorganic & Medicinal Chemistry 46: 116364. 10.1016/j.bmc.2021.116364. 34450570 PMC8354861

[vms371073-bib-0018] Zuzzi‐Krebitz, A. M. , K. Buchta , M. Bergmann , et al. 2024. “Short Treatment of 42 Days With Oral GS‐441524 Results in Equal Efficacy as the Recommended 84‐Day Treatment in Cats Suffering From Feline Infectious Peritonitis With Effusion‐A Prospective Randomized Controlled Study.” Viruses 16: 1144. 10.3390/v16071144. 39066306 PMC11281457

